# Veterinary Science, Important to the Agricultural Interests of Pennsylvania and the United States Generally

**Published:** 1818

**Authors:** James Carver

**Affiliations:** Veterinary Surgeon; Philadelphia


### VETERINARY SCIENCE,

### IMPORTANT

TO THE

### *Agricultural Interests of Pennsylvania;*

AND THE

### UNITED STATES GENERALLY:

Giving an Account of the

### ROYAL VETERINARY COLLEGE OF LONDON,

AND

*A Similar Establishment now Organizing in this City,*

OF

### A New Veterinary Forge and Horse Infirmary,

FOR

### INSTRUCTING CITY AND COUNTRY SMITHS

In that Important Branch of Domestic Science, called

### THE SHOEING ART.

ALSO,

### COMPARATIVE SHOEING EXPLAINED;

Descriptive of a Plan for Disseminating this Necessary and Useful branch of Science

### THROUGHOUT THE UNITED STATES.

BY J. CARVER, *Veterinary Surgeon.*

*To the honourable Judge Peters, Dr. Caldwell, Dr. James, Dr. Cox, Dr. Chapman, Dr. Hewson, General Cadwallader, Roberts Vaux, Reuben Haines, Jeremiah Warder, William Sansom, John Tomlinson, and Dr. Shaw.*

GENTLEMEN,

DEEMING you so justly entitled to the first tender of an essay of this sort, from the station many of you hold in public life, and the estimation which all your private characters bear, that whatever presumption there may appear to be, in making it without permission, I flatter myself that I cannot easily be censured for the apparent impropriety of this address.

I cannot forbear offering some remarks respecting the Veterinary profession, independent of the subject of the new Veterinary Forges I am now establishing in this city, with a view of laying the foundation stone for ameliorating the Diseases of Quadrupeds in general, and in order to give encouragement to those who may still feel a want of confidence in not knowing its having assumed the form of a science, and to the public generally, who have hitherto withheld their support, as if it were a derogatory and hopeless profession. There is no art, it may be maintained, so perplexed and difficult, that by human industry and research, steadily and properly exerted, cannot be rendered more clear and practicable; to accomplish this, however, time must be allowed. Having now for some months past commenced practice, and having performed several successful cures in an *epidemic disease*, which made its appearance in this city during last winter; the result of those inquiries being now before you, the progress no doubt will be more rapid, and its service to the horse and to mankind will assuredly be felt.

Some disappointment has, without doubt, arisen, from unfounded expectations of relief in desperate and hopeless cases, where human art could not avail; and some, not finding their interest served in this respect, have become rancorous enemies to the establishment of the New Forges, as well as to the profession. "The *fruit* has been sought before the *blossom* was unfolded." Still there can be no doubt, that if human medicine and surgery have been aided by public establishments, the Veterinary art must admit of improvement by the same means; and that cloud of *imbecility* that has so long *obscured and stigmatized* the profession in this country, now promises gradually to be dispelled; and no doubt that in a few years, there will not be a city, a town, or a country village in the United States, but will have to boast a practitioner, whose abilities may do honour to a great national institution; and if any exertions on my part, as a fellow citizen, should have been devoted to obtain this useful and desirable end, I feel already the pleasing consolation that I shall die happy, in having done my duty.

I cannot, however, conclude, without the satisfaction of recording the many marks of friendship and assistance shown to me by Jeremiah Warder, Reuben Haines, the late Dr. Rush, General Cadwallader, Judge Peters, and others, on the entrance of my professional studies, at my departure for the Veterinary College. The services of these friends, with pleasure I acknowledge, have been most gratifying to me.

I cannot, at the same time, fail to mention the friendly assistance of Messrs. G. Morrison, Wurts, and Ely, of Boston, and others of this city, for their friendly aid, while at college, at a period when it was most wanted and felt. And not less, also, do I feel indebted to the friendship of Doctors Chapman and Hewson, for their professional and friendly support and cheerful compliance in attending the examination of the death of Mr. Chancellor's horses. Not forgetting my worthy and particular friend Mr. C. Watson, since my return to the city.

I have long foreseen, gentlemen, the innumerable difficulties which still accumulate before me; I feel myself, however, sufficiently bold to encounter them; and although the task which my present situation imposes on me is great, no exertions on my part shall be wanting. Still, to exert every ability to fulfil it, and if the public which now honour me with its confidence, shall continue to encourage this well-meant endeavour to alleviate the sufferings of the *brute creation*, I shall also make every exertion to correspond with their candour, by rendering myself useful in my station.

Some of you, gentlemen, are proprietors of a very considerable stock of cattle; and the many different maladies and disorders, to which the whole species are particularly exposed and subject, and which, consequently, in gratitude, require our friendly aid and assistance; and as this branch of domestic science, called the Veterinary Art, which I have now the honour to profess among you, has stood so preeminently useful in your opinions, I flatter myself that the following sheets will not be an unacceptable token of respect. All I mean by this attempt, is to endeavour to be useful.

I only desire Mr. Pope's rules may be observed:

"In every work regard the writer's end,
Not free from fault, nor yet too vain to mend."

And should this imperfect performance; for want of a better, prove commendable to society, by these few hints which I have drawn together, I shall, with pleasing satisfaction, reflect, that I have endeavoured to contribute my mite towards the benefit of mankind, as well as the brute creation.

I have the honour to be,

Gentlemen, your respectful

And obedient servant,

J. CARVER.

Philadelphia, August, 1818.

